# ^89^Zr-mAb uptake interpretation requires the use of tissue to plasma ratios corrected for antibody catabolism

**DOI:** 10.1186/s13550-025-01315-6

**Published:** 2025-09-26

**Authors:** Marc C. Huisman, Johanna E. E. Pouw, Sandeep S. V. Golla, Damien Huglo, Franck Morschhauser, Josée M. Zijlstra, Jessica E. Wijngaarden, Mirte Stavenga, Hylke J. Sebus, Maarten Slebe, Andrea Thiele, Danielle Vugts, Iris H. C. Miedema, Gerben J.C. Zwezerijnen, Idris Bahce, C. Willemien Menke-van der Houven van Oordt, Dhaval K. Shah, Yvonne W. S. Jauw, Ronald Boellaard

**Affiliations:** 1 Department of Radiology and Nuclear Medicine, Amsterdam UMC, Amsterdam, The Netherlands; 2 Cancer Center Amsterdam, Imaging and Biomarkers, Amsterdam, The Netherlands; 3 Department of Medical Oncology, Amsterdam UMC, Amsterdam, The Netherlands; 4 Department of Hematology, EA7365-GRITA-Groupe de Recherche sur les forms Injectables et les Technologies Associées, Université de Lille, CHU Lille, Lille, France; 5 Department of Hematology, Amsterdam UMC, Amsterdam, The Netherlands; 6 Department of Pulmonary Medicine, Amsterdam UMC, Amsterdam, The Netherlands; 7 Department of Translational Medicine & Clinical Pharmacology, Boehringer Ingelheim Pharma GmbH & Co. KG, Biberach an der Riss, Germany; 8 Department of Pharmaceutical Sciences, School of Pharmacy and Pharmaceutical Sciences, The State University of New York at Buffalo, Buffalo, USA

**Keywords:** ^89^Zr-mAb, ^89^Zr-immuno-PET, Quantification

## Abstract

**Background:**

Typically, tissue uptake, expressed in tissue over plasma ratio (TPR), is reported from a ^89^Zr-mAb PET study. Nonspecific antibody catabolism and the residualizing property of ^89^Zr hamper the use of tissue uptake as a measure for target mediated uptake. Therefore, we propose to report uptake as tissue over plasma ratio additionally corrected for nonspecific antibody catabolism (cTPR). We introduce an organ specific but time independent baseline tissue over plasma ratio (bTPR), which is the expected tissue over plasma ratio in the absence of target mediated uptake. Target mediated uptake is then evident from a cTPR value higher than bTPR. We analyzed retrospectively tissue uptake reported for three ^89^Zr-mAb studies. The method of correction for non-specific uptake due to antibody catabolism requires the net rate of irreversible ^89^Zr uptake due to nonspecific antibody catabolism, total tissue exposure to ^89^Zr-mAb (the area under the plasma time concentration curve, obtained from blood sampling) and a single PET scan taken more than 24 h post injection.

**Results:**

For ^89^Zr-Cetuximab, 38% of spleen uptake at 144 h post injection is due to nonspecific antibody catabolism (median, *n* = 7). Furthermore, it ranges between 27 and 63% due to interindividual variability in tissue exposure. Patients are ranked differently for total compared to corrected ^89^Zr-mAb uptake, leading to the risk of invalid conclusions from PET studies if based on total uptake.

**Conclusion:**

^89^Zr-mAb PET uptake should be reported as tissue over plasma ratio corrected for nonspecific antibody catabolism.

**Supplementary Information:**

The online version contains supplementary material available at 10.1186/s13550-025-01315-6.

## Background

**Fig. 1:**
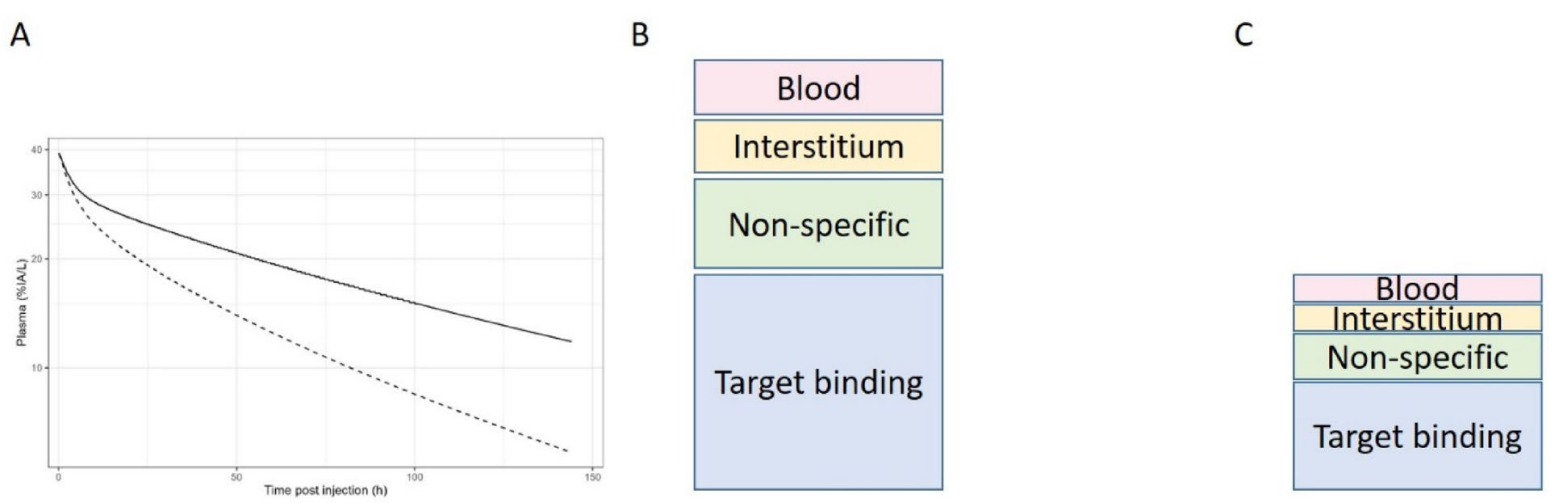
Schematic overview of contributions to measured uptake for the same tracer in a situation with slow kinetics (at high mass dose, solid line in panel **A** and panel **B**) and with fast kinetics (at low mass dose, dashed line in panel **B** and panel **C**)

To study biodistribution and tumor uptake of monoclonal antibodies (mAb) we can label them with ^89^Zr and perform a ^89^Zr-mAb PET study. Typically, we report and interpret total tissue uptake as total tissue mAb concentration, derived from the PET signal and reported as standardized uptake value (SUV) or %injected activity. However, as has been shown previously, SUVs may not be a valid readout for target mediated uptake in case of non-linear plasma kinetics, i.e. the clearance of mAb and ^89^Zr-mAb are not proportional to the injected mass due to fast uptakes in tissues or plasma binding, particularly occurring at very low injected masses (typically at mass doses < 50 mg), see also Fig. 1.

Consequently, SUVs are not a valid readout for target mediated uptake under these conditions as SUVs are based on the assumption that tissue exposure (or plasma kinetics) scale proportionally to injected activity over weight. Wijngaarden et al. [1] has shown that tissue to plasma ratios are more accurate simplified metrics to assess total tissue uptake validated against quantitative kinetic analysis using Patlak linearization. Secondly, even when using TPR the interpretation of ^89^Zr-mAb uptake is hampered by non-target mediated uptake, caused by nonspecific antibody catabolism, followed by intracellular residualization of ^89^Zr. The aim of this paper is to illustrate the need for using TPR that are additionally corrected for non-specific antibody catabolism to theoretically allow for a more accurate interpretation of PET signals. After a discussion of non-specific antibody catabolism the correction method is illustrated. Currently, knowledge on the determinants of the net rate of irreversible uptake due to non-specific catabolism (e.g. antibody clones, disease characteristics or inter individual variability) is very limited. Therefore, the practical applicability of the correction method will need further validation, and a way of validation will be outlined.

## Methods

### Theoretical background

**Fig. 2:**
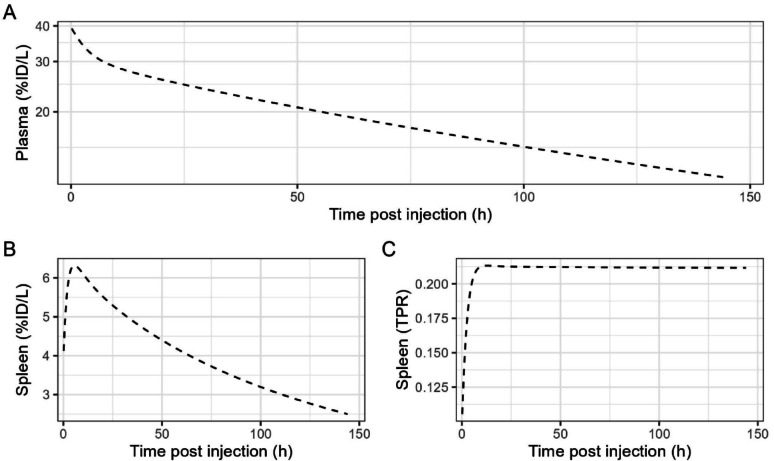
Schematic overview of PBPK predicted mAb concentrations in the absence of target expression in plasma (panel **A**) and spleen (panel **B** and **C**), given as percentage of the injected mAb dose per liter (panel **A** and **B**) or as tissue over plasma ratio (panel **C**)

The mechanisms of mAb distribution into and clearance from tissue are well described in literature [2–4], enabling a physiologically based pharmacokinetic (PBPK) modeling prediction of plasma and tissue concentration after intravenous injection of mAb [5]. In the absence of target expression, mAb concentrations in plasma and spleen as a function of time are given schematically in Fig. 2.

After equilibration, for a tissue without relevant expression of the target antigen (which we typically assume to take up to 24 h [6]), the concentration of mAb in tissue is predicted to become proportional to the concentration of mAb in plasma, with the proportionality constant referred to as the antibody biodistribution coefficient (see panel C, where the spleen concentration in TPR becomes constant [6]). It reflects the fact that the concentration of mAb in tissue is the sum of the concentrations of mAb in vascular and in interstitial space, both proportional to the plasma concentration. The concentration in tissue interstitial space is proportional to the plasma concentration as the continuous uptake from vascular space is in equilibrium with clearance via the lymphatic system and antibody catabolism following nonspecific pinocytosis.

**Fig. 3:**
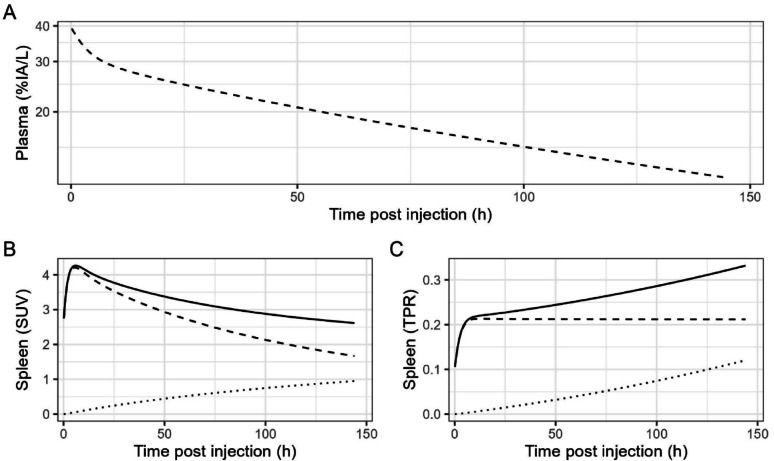
Schematic overview of predicted ^89^Zr-mAb uptake in the absence of target expression in plasma (panel **A**) and spleen (panel **B** and **C**). Total uptake (solid lines) is the sum of two components: one due to ^89^Zr linked to mAb (dashed lines), the other due to residualization of ^89^Zr after nonspecific antibody catabolism (dotted lines). Uptake is given as percentage of the injected activity per liter (panel **A**), as standardized uptake value (panel **B**) or as tissue over plasma ratio (panel **C**)

A PET measurement consists of measuring tissue concentrations of a radiolabeled compound in Bq/mL, which we call uptake and typically present as either standardized uptake value (SUV; concentration divided by the injected activity per kg body weight) or tissue over plasma ratio (TPR; tissue concentration divided by the concurrent concentration in plasma). Thus, our prediction for ^89^Zr-mAb uptake in plasma and spleen in the absence of target expression is given with the dashed lines in Fig. 3. For plasma (panel A), the curve is similar to panel A of Fig. 2, with the concentration given in %injected activity per L instead of %injected dose per L. Assuming a body weight of 80 kg, we obtain the dashed curve in panel B. This is the same as the curve in panel B of Fig. 2, scaled by a factor of 80 (kg)/100(%) to convert the uptake measure %ID/L into SUV. The dashed line in panel C is similar to the dashed line in panel C of Fig. 2, as the uptake unit is also TPR.

As a result of the residualizing property of ^89^Zr, it is trapped intracellularly after nonspecific antibody catabolism [7–10]. The total concentration of residualized ^89^Zr is driven by total tissue exposure (quantitatively equal to the area under the plasma concentration time activity curve between the time of injection and the timepoint under consideration), and increases with time.

Previously, nonspecific antibody catabolism was assessed in tissues without target [11]. By using Patlak linearization the net rate of irreversible uptake due to nonspecific antibody catabolism was found to be tissue dependent and in the order of 0.1-1 µL/g/h. Baseline *V*_*T*_ values were derived and found to be in the order of 0.1–0.25 mL/cm^3^.

Here we use this result to analyze uptake measured from a single scan, acquired later than 24 h post injection. The method uses patient specific total tissue exposure values to calculate and subtract uptake due to nonspecific antibody catabolism. The resulting uptake, divided by the plasma concentration, equals the corrected TPR value (cTPR). If a cTPR value is higher than the tissue specific baseline TPR value (bTPR) we can conclude on the presence of target mediated uptake in this tissue.

### Correction for nonspecific antibody catabolism

We base the correction for nonspecific antibody catabolism (NAC) on recent work that quantified ^89^Zr uptake in tissues without target expression [11]. Measured uptake was shown to be consistent with the assumptions of the Patlak linearization [12], and could therefore be written as the sum of a reversible and an irreversible part:

1 $$\:{C}_{tissue}=\:{V}_{T,\:tissue}{C}_{plasma}+\:{K}_{i,tissue}{AUC}_{plasma},$$

where *C*_*tissue*_ (in Bq·mL^−1^) and *C*_*plasma*_ (in Bq·g^−1^) are the measured ^89^Zr concentration in tissue and plasma, respectively and *AUC*_*plasma*_ (in Bq·ml^−1^·h) is total tissue exposure. This approach introduces two constants; the distribution volume *V*_*T, tissue*_ (in mL·cm^−3^) and the net rate of irreversible uptake *K*_*i, tissue*_ (in µL·g^−1^·h^−1^). Known values obtained for tissues with mAb that do not show target mediated uptake in the tissue are summarized in Table 1.

**Table 1:** Tissues for which values of V_T_ and K_i_ in the absence of target expression are known

	V_T_ (in mL/cm^3^) median (IQ range)	K_i_ (in µL·g^−1^·h^−1^) median (IQ range)	mAb used to derive baseline	Reference
Bone Marrow	N.A.	N.A. (0.2–0.8)^*^	BI 754,111, Durvalumab Nivolumab, Pembrolizumab, Ipilimumab	[13]
Brain	N.A.	N.A. (0.0-0.1)^*^	BI 754,111, Durvalumab Nivolumab, Pembrolizumab, Ipilimumab	[13]
Kidney	0.20 (0.16–0.25)	0.7 (0.4–1.3)	Cetuximab, Obinituzumab, Trastuzumab, Hu-J591	[11]
Liver	0.24 (0.21–0.28)	1.1 (0.8–2.1)	Cetuximab, Obinituzumab, Trastuzumab, Hu-J591	[11]
Lung	0.09 (0.07–0.10)	0.2 (0.1–0.3)	Cetuximab, Obinituzumab, Trastuzumab, Hu-J591	[11]
Spleen	0.24 (0.20–0.27)	0.5 (0.3–0.7)	Cetuximab, Obinituzumab, Trastuzumab, Hu-J591	[11]
Tumor	0.24^**^	N.A. (0.4–0.9) ^*^	Rituximab 89Zr-CEA-IL2v	[14]

*These ranges are full, not IQ ranges.**This is a calculated value [15]

Here, we interpret the *K*_*i, tissue*_ values from Tabel 1 as *K*_*NAC, tissue*_ (the observed value for the net rate of irreversible uptake due to NAC). Similarly, *V*_*T, tissue*_ is interpreted as *bTPR*_*tissue*_ (the observed baseline value for the tissue over plasma ratio in the absence of target expression).

Uptake due to NAC is equal to the product of *K*_*NAC, tissue*_ and total tissue exposure to ^89^Zr-mAb ($\:{AUC}_{plasma}$):

2 $$\:{C}_{NAC}=\:{K}_{NAC,tissue}{AUC}_{plasma}.\:$$

In the absence of target expression, we expect that after subtraction of *C*_*NAC*_ and division by $\:{C}_{plasma}$ we obtain from Eq. (1):

3 $$\:{cTPR}_{tissue}=\:\frac{\left({C}_{tissue}-{C}_{NAC}\right)}{{C}_{plasma}},$$

with *cTPR* the tissue over plasma ratio after total tissue uptake is corrected for NAC.

For practical implementation, we obtain *C*_tissue_ from a volume of interest delineated on a PET scan acquired later than 24 h post injection and the concentration of ^89^Zr in plasma at the time of the scan. In addition, we need the net rate of irreversible uptake due to NAC (from Table 1) and the patient specific total tissue exposure (from blood sampling). We subtract $\:{C}_{NAC}$ from *C*_tissue_ and divide by $\:{C}_{plasma}\:$to obtain *cTPR*_tissue_. The presence of target mediated uptake is assessed by comparing $\:{cTPR}_{tissue}$ with $\:{bTPR}_{tissue}$. Table 2 lists the studies that are used in the analysis.

**Table 2:** Information on the 89Zr-immuno-PET studies used in this paper

mAb	Obinutuzumab	Cetuximab	Durvalumab
Target	CD20	EGFR	PDL1
# of patients	9	7	5
ID (mg) labeled + unlabeled	10 + 1,000	10 + 500 mg/m2	0.15 + 22.35
Scheduled PET scan times (h)	1, 72 and 144	1, 72 and 144	41, 110, 162
Scheduled sample times (h)	0.08, 0.5, 1, 2, 24, 72, 144	1, 24, 48, 72, 144	0.2, 0.7, 1, 2, 41, 110, 162
References	[17]	[18]	[19]

R 4.4.0 (R Core Team) was used to compute *p* values from two-sided t-tests (for uptake values versus *bTPR*_*tissue*_ or for uptake values between two groups).

## Results

**Fig. 4:**
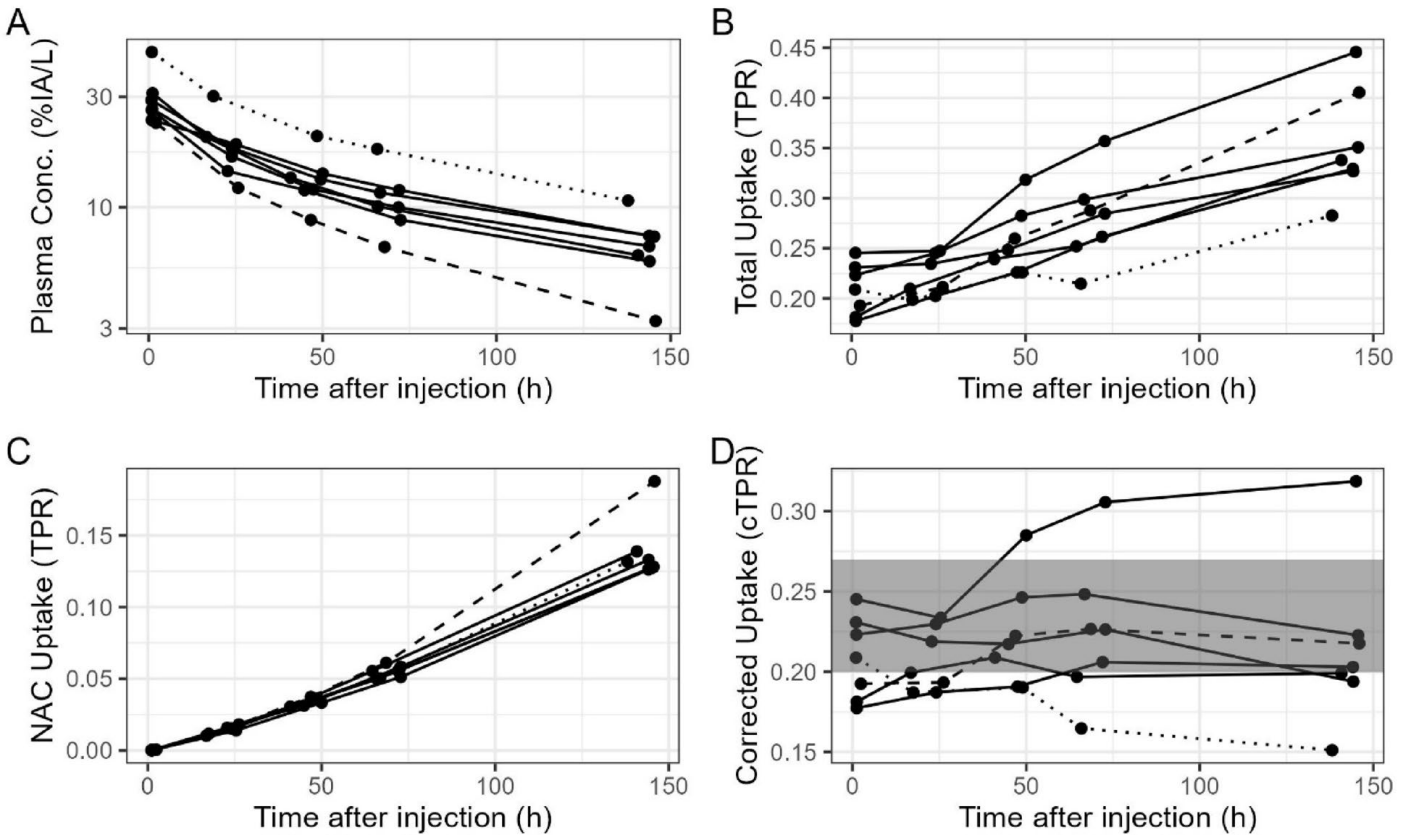
Measured ^89^Zr-cetuximab concentration in plasma (panel **A**, in %IA/L), total measured ^89^Zr-cetuximab spleen uptake (panel **B**, in TPR), calculated uptake due to nonspecific antibody catabolism of ^89^Zr-cetuximab in the spleen (panel **C**, in TPR) and nonspecific antibody catabolism corrected ^89^Zr-cetuximab spleen uptake (panel **D**, in cTPR). The gray area in panel **D** indicates the range of baseline TPR_spleen_ values from Table 1. Each line represents data from a single patient. The dotted and dashed lines correspond to data from the patient with highest and lowest tissue exposure, respectively

Measured ^89^Zr-cetuximab plasma pharmacokinetics is given as %IA/L in panel A of Fig. 4 (please note the logarithmic scale). Every line represents data from a single patient. The dotted and dashed lines correspond to data from the patient with highest and lowest tissue exposure, respectively. Total measured ^89^Zr-cetuximab spleen uptake in TPR is given in panel B (median TPR at 144 h post injection 0.34, range 0.28–0.45; see Supplemental Fig. 2–4 for uptake in kidney, liver and lung).

**Fig. 5:**
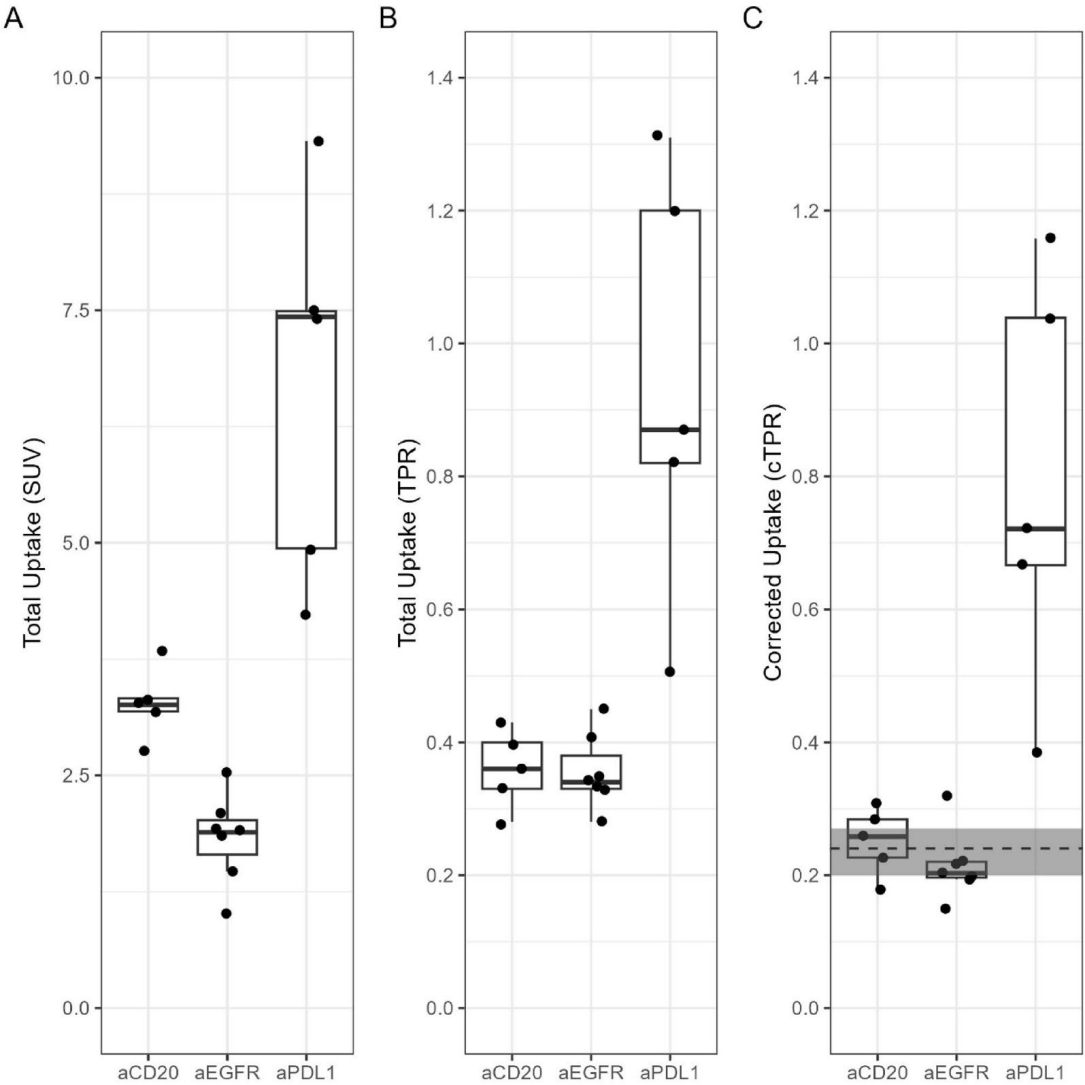
Total spleen uptake in SUV (panel **A**), in TPR (panel **B**) and nonspecific antibody catabolism corrected uptake in cTPR (panel **C**) at the last imaging timepoint (6-7 days post injection). The gray area in panel **C** indicates the range of baseline TPR_spleen_ values from Table 1

Tissue uptake due to nonspecific antibody catabolism (median TPR at 144 h post injection 0.13) is directly driven by exposure (Eq. 2) and plotted as TPR in panel C of Fig. 4. The variation in exposure (the lowest exposure indicated with a dashed and the highest by a dotted line in panel A) translates into a factor of 1.5 between lowest and highest uptake due to nonspecific antibody catabolism in panel C (at 144 h post injection the TPR values are 0.13 and 0.19, respectively). Nonspecific antibody corrected uptake is given as cTPR in panel D. Most cTPR values are within the range of bTPR values from Table 1. The subject with highest tissue exposure (data of which are given with dotted lines in Fig. 4) shows also highest uptake due to nonspecific antibody catabolism, as expected (although this cannot be readily seen in panel C of Fig. 4 as nonspecific uptake is given there as TPR). It is not the subject with highest total uptake, indicating that not only exposure drives total uptake. Additional contributions may be due to variability in patient biology (vascular volume fraction, available interstitial space volume or off-target interaction with e.g. Fcγ receptors). After correction for nonspecific antibody catabolism, this subject shows the lowest uptake for all patients in the group. The subject with lowest tissue exposure (data indicated with a dashed line) shows, as expected, lowest uptake due to nonspecific antibody catabolism and also lowest total uptake. After correction for nonspecific antibody catabolism the uptake for this patient is average compared to the other patients in the group. In conclusion, the ranking of total uptake is not the same as the ranking for nonspecific antibody catabolism corrected uptake (See Suppplemental Fig. 1 for the correlation of TPR and cTPR at the latest imaging time point). Uptake at the last imaging time point (144–162 h post injection) is given in Fig. 5. Panel A shows total uptake in SUV, panel B total uptake in TPR and panel C shows nonspecific antibody catabolism corrected uptake. Before correction, total uptake for ^89^Zr-cetuximab differs from ^89^Zr-obinutuzumab uptake (median SUV 3.32, range 3.11–4.39, *p* < 1e5), although this is not expected as there is no target expression [11]. A part of the difference in total uptake is due to the different plasma pharmacokinetics: for ^89^Zr-obinutuzumab, total tissue exposure at the last imaging time point (144 h post injection) is 0.027 (median; range 0.021–0.035) h/mL, whereas for ^89^Zr-cetumimab total tissue exposure at the same scheduled imaging time point is 0.017 (range 0.012–0.028) h/mL. After correction, uptake data are not different from bTPR (median cTPR 0.23, range 0.15–0.35, *p* = 0.72 and median 0.20, range 0.15–0.32, *p* = 0.25, respectively), and they do not differ anymore (*p* = 0.53), as expected [11]. However, corrected ^89^Zr-durvalumab uptake is above baseline, indicating target mediated uptake in the spleen for this mAb, as expected.

## Discussion

As a consequence of nonspecific antibody catabolism, which is thought to occur in all tissues throughout the body, and the residualizing property of ^89^Zr, measured ^89^Zr-mAb-PET uptake always increases with time post injection, even in the absence of target-specific binding (see Fig. 3). This paper discusses the consequences of nonspecific antibody catabolism on the interpretation of measured ^89^Zr-mAb-PET uptake.

For the spleen, nonspecific antibody catabolism contributes a median SUV of 0.7 and TPR of 0.13 for ^89^Zr-cetuximab uptake at 6 days post injection. From Table 1 it can be concluded that nonspecific antibody catabolism contributes to SUV with a median value that ranges between 0.3 and 1.5 for brain and liver, respectively. For the spleen, a significant and variable SUV uptake range due to nonspecific antibody catabolism of 0.5-1.0 is observed. This is not unexpected, as significant inter individual variability in plasma PK is often observed, even at mAb mass doses that result in linear PK [19, 20]. Although the amount of uptake due to nonspecific antibody catabolism increases with time, uptake in terms of TPR after correction for nonspecific antibody catabolism is constant at imaging times after equilibration of the biodistribution (see Fig. 3). The fact that the ranking in corrected uptake over the various patients in the ^89^Zr-cetuximab study is different from the ranking in total uptake suggests that interpretation of total uptake data in relation to clinical benefit or IHC based target expression may be hampered by interindividual variability in tracer exposure. Improved understanding would require correction for nonspecific antibody catabolism.

Correlation of cTPR (a measure of target specific binding) with an ex-vivo standard of target expression would be very beneficial. However, we do not know of a dataset that would allow to describe that correlation. A main reason is that a correlation between target specific binding and ex-vivo IHC is hampered by the different volumes that are sampled (much smaller for IHC than for PET). This lack of data results in a limitation of this study, which theoretically shows why cTPR should be preferable to SUV or %IA/L as measure of target specific binding, but requires more validation that this results in a more accurate interpretation of specific uptake.

Obtaining knowledge on tracer exposure requires blood sampling. For a new ^89^Zr-mAb to be used clinically, a pilot study may be set up that includes blood sampling and imaging at a single late time point. A standardized sampling interval (at 5–10, 30, 60 and 120 min post end of injection and at 72 ± 8 and 144 ± 8 h) would allow to build a larger database of exposure data that will help in better understanding variability in the contribution of nonspecific antibody catabolism to total ^89^Zr-mAb PET uptake. In addition, the correction for nonspecific antibody catabolism requires a priori knowledge of the net rate of irreversible binding due to nonspecific antibody catabolism in the tissue of interest.

The use of the earlier derived *V*^*t*^ and *K*^*i*^ values as general parameters in a correction for nonspecific antibody catabolism implicitly assumes that they are tracer independent, as well as non-sensitive to specific antibody class, patient or disease characteristics. We have not been able to test this hypothesis because we lack the necessary data. The approach is a pragmatic one, based on the fact that the rate of nonspecific antibody catabolism is determined by the rate of nonspecific pinocytosis and the kinetics of binding to FcRn; these may be constant for a large class of mAb. The inter-patient variation in nonspecific antibody catabolism induced by changes in plasma concentration of ^89^Zr-mAb is taken into account. Therapeutic antibodies in clinical trials are typically modified for decreased FcγR interaction [21], which is therefore assumed to play a minor role. As these interactions are not directly translatable between mice and men, preclinical data may be limitedly helpful in finding the magnitude of factors potentially influencing baseline uptake.

The lack of information on the net rate of irreversible uptake due to non-specific catabolism currently forms a limitation for practical applicability of the correction method. Validation of the method can be done for tissues that can be assumed to be effectively without target expression (either because the target is not present in the tissue or because the target can be saturated by a sufficiently high unlabeled dose of mAb or by another blocking agent or mAb that blocks the specific binding target). In such a situation cTPR should be within the baseline range (as in Fig. 4D). If this is not the case, the net rate of non-specific uptake should be adapted to ensure that the resulting cTPR values are within the baseline range (for all measurement time points larger than 24 h post injection). For any (new) mAb validation of the applicability of the proposed methods and/or reference values should be validated.

It is important to realize that study design can have a significant impact on the ability to detect target engagement [11]. For example, for ^89^Zr-anti-CD20, no increase in irreversible uptake (*K*_*i*_) was observed in the spleen and liver, although expression of CD20 is known to be high [22, 23]. A plausible explanation for this observation is saturation of the CD20 receptors due to a high dose of unlabeled anti-CD20 (1,000 mg), which was administered before the radiolabeled antibody. So, antibody mass dose is a parameter to be considered in assessment of target engagement.

For tissues where no *K*_*i*_ value is known (or for a tissue were the *K*_*i*_ value may be different due to specific characteristics or modifications of the ^89^Zr-labeled mAb) the correction method outlined here can be used to derive it, if one can assume the absence of target specific binding under the conditions during the imaging study. Based on published antibody biodistribution coefficient values and blood volume fractions [11], bTPR value can be predicted, allowing interpretation of the measured cTPR value for different ^89^Zr-labeled mAb in that tissue in terms of target engagement. Thus, the cTPR values for ^89^Zr-durvalumab in the spleen being higher than bTPR constitutes experimental evidence for target mediated uptake in the spleen, as observed earlier [13]. In this case, the cTPR value will also increase with time.

The ^89^Zr-aEGFR and ^89^Zr-aCD20 data used here were also used in the derivation of bTPR (together with data on ^89^Zr-aHER2 and ^89^Zr-aPSMA; [11]). Therefore, the fact that cTPR values are in line with bTPR was to be expected. The calculation here, however, does only require a single PET scan and does only rely on the assumptions underlying the Patlak approach for the subtraction of nonspecific antibody catabolism, not for total measured tissue uptake.

In this manuscript we only consider ^89^Zr-mAb catabolism as source of free ^89^Zr in the body (this can take place in tissues as well as in PBMCs). In this case, catabolism will be followed by intracellular residualization of ^89^Zr. Free ^89^Zr in plasma is expected to play a minor role, as ^89^Zr-mAb metabolizes very slowly (it is expected that more than 95% of the concentration of ^89^Zr mAb in plasma is in the form of ^89^Zr-mAb at 6 days post injection [24]). A potential source of free ^89^Zr in blood is direct injection as part of the tracer. This ^89^Zr is thought to be in the form of radiometal not associated with the chelator, due to instability of the hexadentate ^89^Zr chelator complex predominantly used in PET studies. In clinical studies, the tracer purity is strictly regulated and the percentage free ^89^Zr is therefore minimal. Free ^89^Zr in blood is expected to be renally cleared rapidly [10].

Typically, a late imaging timepoint is used for visual interpretation of ^89^Zr-mAb PET scans: plasma concentration decreases and uptake in tissues and tumors increases, increasing visual contrast for regions with enhanced uptake. Part of this uptake is, however, always also due to nonspecific antibody catabolism which shows significant interindividual variability. Therefore, reporting uptake as a readout for target mediated uptake of a ^89^Zr-mAb PET study in cTPR values to allow for an assessment of target mediated uptake is recommended.

There are certainly situations in which total uptake, expressed as %IA/L or total activity (Bq), is the measure of interest (e.g. in dosimetry). In these cases correction for nonspecific antibody catabolism is not needed.

In summary, SUVs should not be used as shown previously by Wijngaarden et al. [1] and TPRs are the preferred simplified metrics taking into account possible inter- and intra-subject variabilities in tissue exposure (i.e. plasma kinetics). Yet, even when using TPR the total uptake is affected by non-specific uptake resulting from antibody catabolism. Therefore, for an accurate interpretation of TPRs a correction for the contribution of non-specific uptake is warranted as well. In this paper we recommend the use of cTPR or, at least consider bTPR, to better assess target mediated uptake.

## Conclusions

We recommend to report ^89^Zr-mAb-PET uptake as tissue over plasma ratio corrected for nonspecific antibody catabolism, which can be calculated by subtracting uptake due to nonspecific antibody catabolism from total uptake and dividing the remaining uptake by the plasma concentration.

## Supplementary Information

Supplementary Material 1

## Data Availability

The datasets used and/or analysed during the current study are available from the corresponding author on reasonable request.
