# Supplementary material for: 89Zr-mAb uptake interpretation requires the use of tissue to plasma ratios corrected for antibody catabolism
Source: EJNMMI Res. 2025 Sep 26;15:122. doi: 10.1186/s13550-025-01315-6 (PMC12474831; doi:10.1186/s13550-025-01315-6)
Supplement: Supplementary file 1 — Supplementary Material 1 [file 13550_2025_1315_MOESM1_ESM.docx]

**^89^Zr-mAb uptake interpretation requires the use of tissue to plasma ratios corrected for antibody catabolism**

European Journal of Nuclear Medicine and Molecular Imaging Research

Marc C. Huisman^*1,2^, Johanna E.E. Pouw^2,3^, Sandeep S.V. Golla^1,2^, Damien Huglo^4^, Franck Morschhauser^4^, Josée M. Zijlstra^2,5^, Jessica E. Wijngaarden^1,2^, Mirte Stavenga^2,3^, Hylke J. Sebus^2,3^, Maarten Slebe^2,6^, Andrea Thiele^7^, Danielle Vugts^1,2^, Iris H.C. Miedema^2,3^, Gerben J.C. Zwezerijnen^1,2^, Idris Bahce^2,6^, C. Willemien Menke-van der Houven van Oordt^2,3^, Dhaval K. Shah^8^, Yvonne W.S. Jauw^2,5^, Ronald Boellaard^1,2^

**Author’s affiliations**

^1^ Department of Radiology and Nuclear Medicine, Amsterdam UMC, Amsterdam, The Netherlands.

^2^Cancer Center Amsterdam, Imaging and Biomarkers, Amsterdam, The Netherlands.

^3^Department of Medical Oncology, Amsterdam UMC, Amsterdam, The Netherlands.

^4^EA7365-GRITA-Groupe de Recherche sur les forms Injectables et les Technologies Associées, Université de Lille, and Department of Hematology, CHU Lille, Lille, France.

^5^Department of Hematology, Amsterdam UMC, Amsterdam, The Netherlands.

^6^Department of Pulmonary Medicine, Amsterdam UMC, Amsterdam, The Netherlands.

^7^Department of Translational Medicine & Clinical Pharmacology, Boehringer Ingelheim Pharma GmbH & Co. KG, Biberach an der Riss, Germany.

^8^Department of Pharmaceutical Sciences, School of Pharmacy and Pharmaceutical Sciences, The State University of New York at Buffalo, Buffalo, USA.

* Corresponding author

**First/corresponding author**

Marc Huisman

Email: [m.huisman@amsterdamumc.nl](mailto:m.huisman@vumc.nl)

**
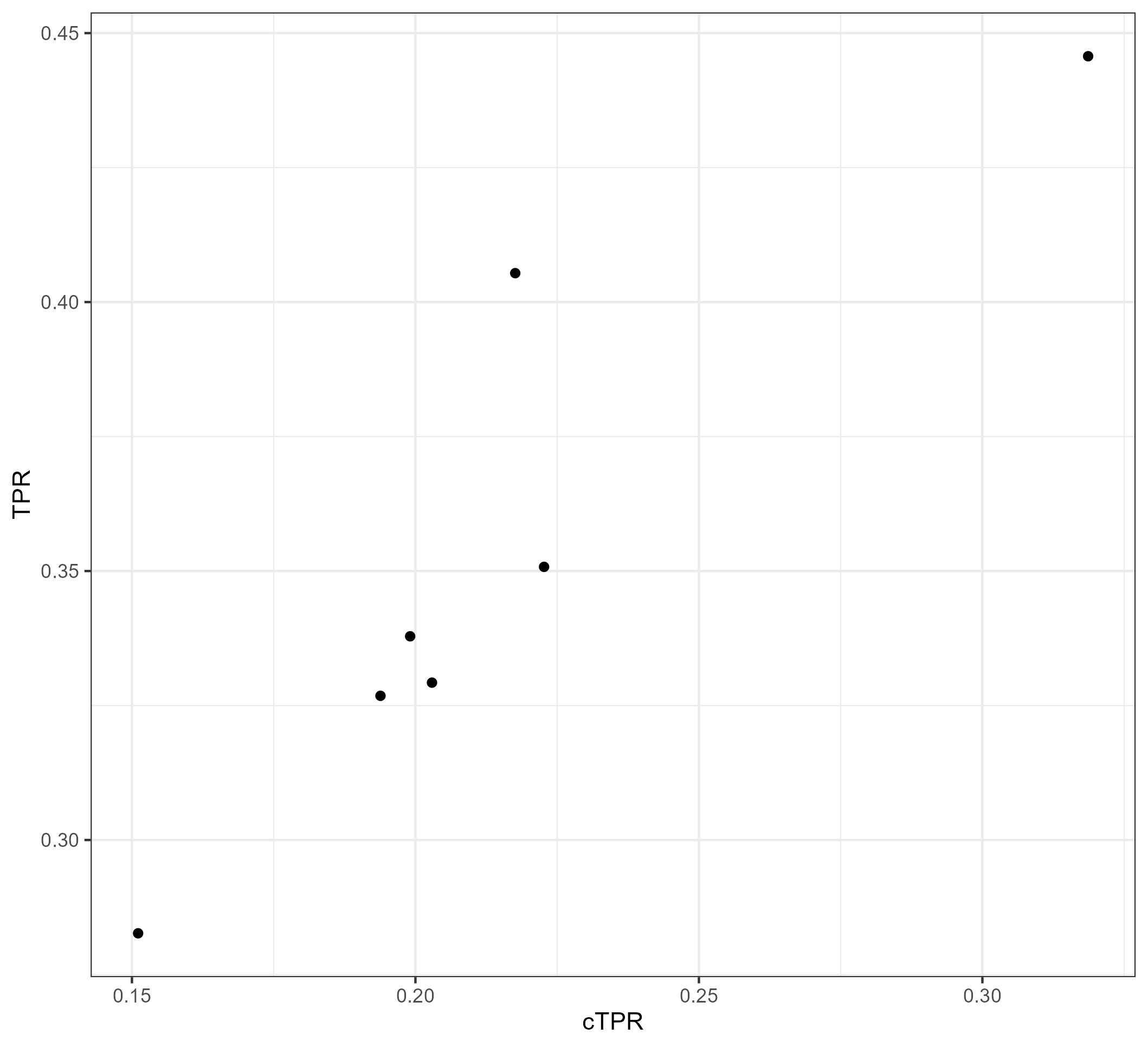
**

**Supplemental Figure 1**. Correlation of TPR and cTPR at the latest imaging time point (six days post injection). For these seven patients, the rank order of 2 of them is different based on TPR than based on cTPR.


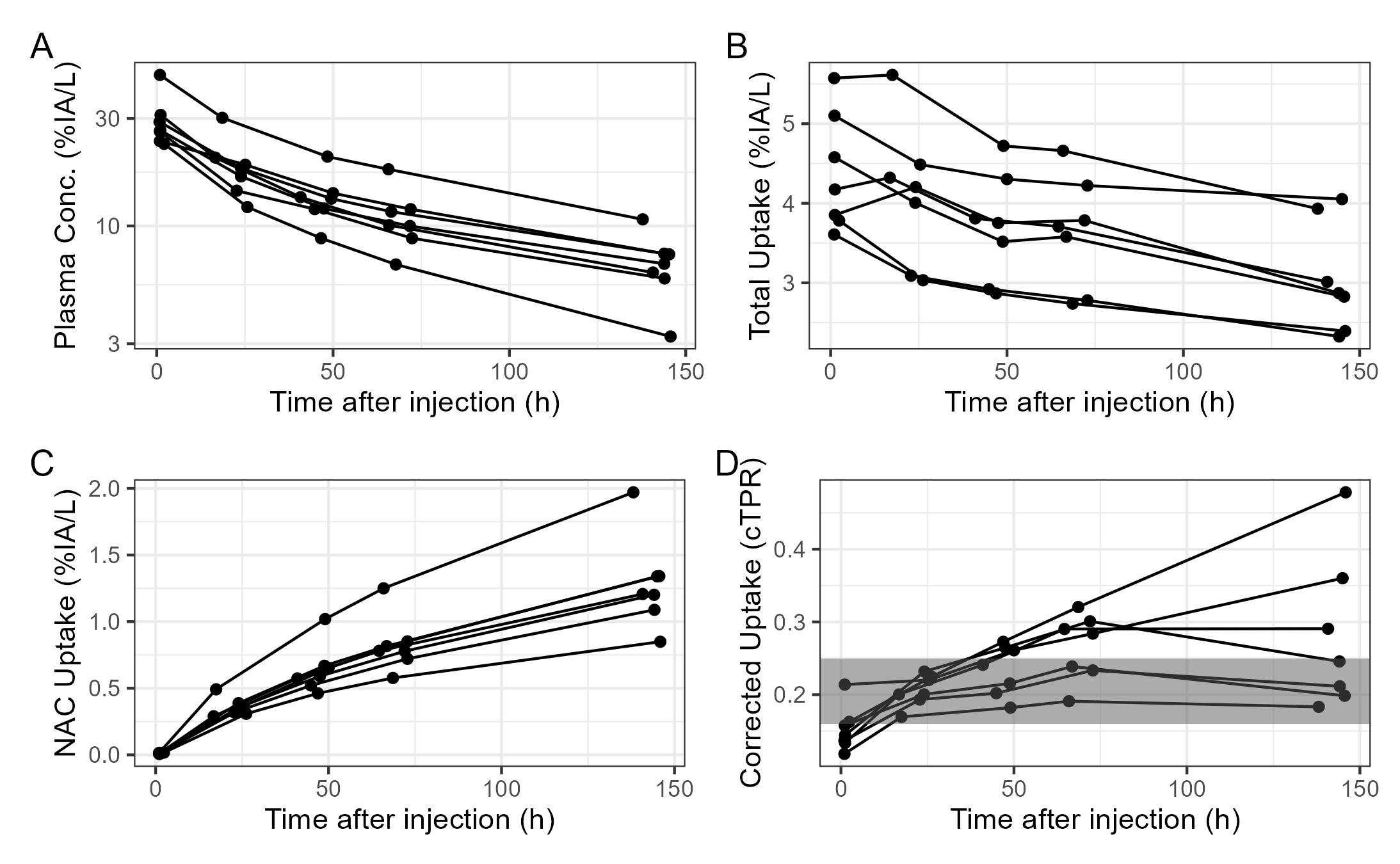


**Supplemental Figure 2**. Measured ^89^Zr-cetuximab concentration in plasma (panel A, in %IA/L), total measured ^89^Zr-cetuximab kidney uptake (panel B, in TPR), calculated uptake due to nonspecific antibody catabolism of ^89^Zr-cetuximab in the kidney (panel C, in TPR) and nonspecific antibody catabolism corrected ^89^Zr-cetuximab kidney uptake (panel D, in cTPR). The gray area in panel D indicates the range of bTPR_kidney_ values from Table 1. Each line represents data from a single patient.


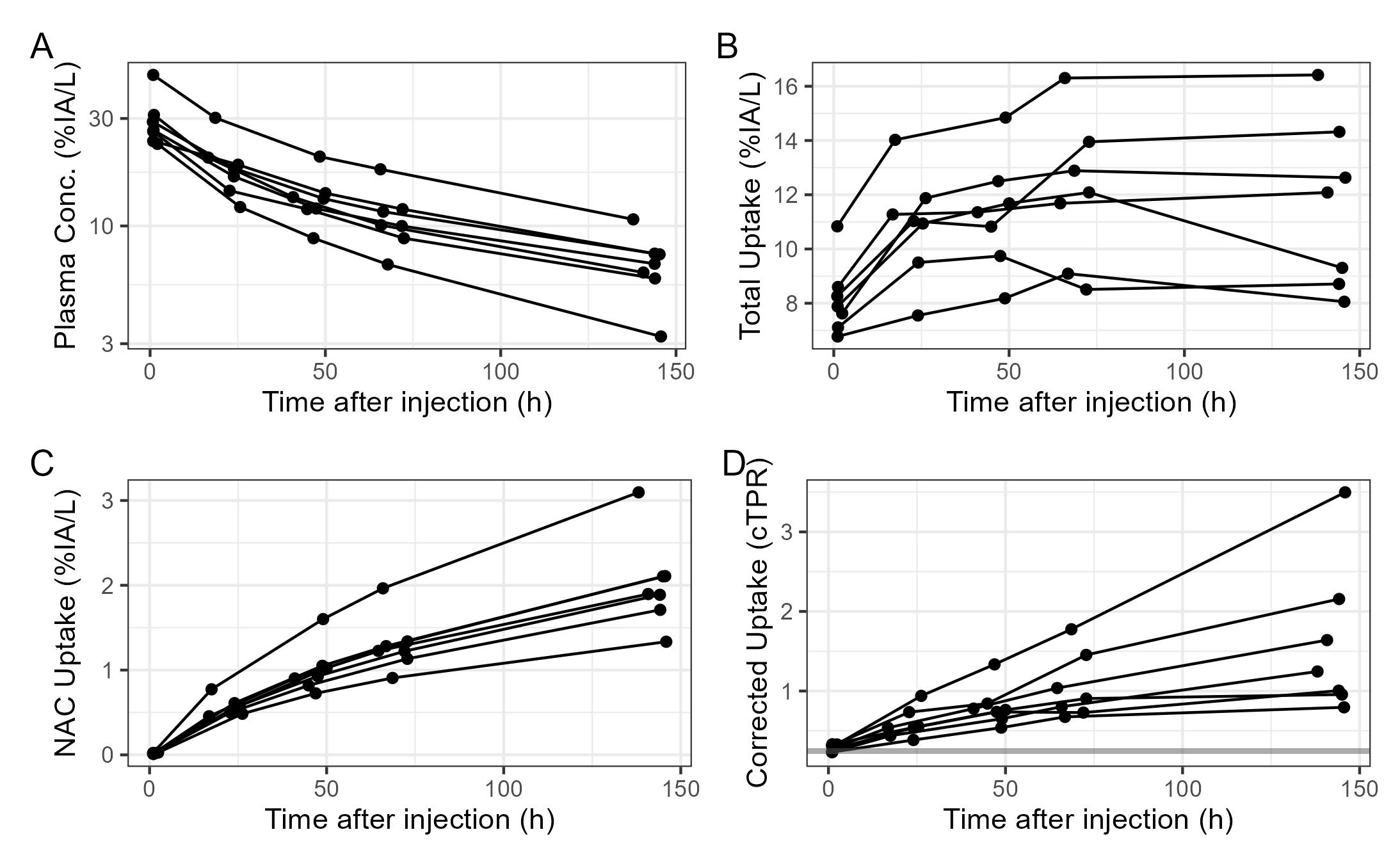


**Supplemental Figure 3**. Measured ^89^Zr-cetuximab concentration in plasma (panel A, in %IA/L), total measured ^89^Zr-cetuximab liver uptake (panel B, in TPR), calculated uptake due to nonspecific antibody catabolism of ^89^Zr-cetuximab in the liver (panel C, in TPR) and nonspecific antibody catabolism corrected ^89^Zr-cetuximab liver uptake (panel D, in cTPR). The gray area in panel D indicates the range of bTPR_liver_ values from Table 1. Each line represents data from a single patient.


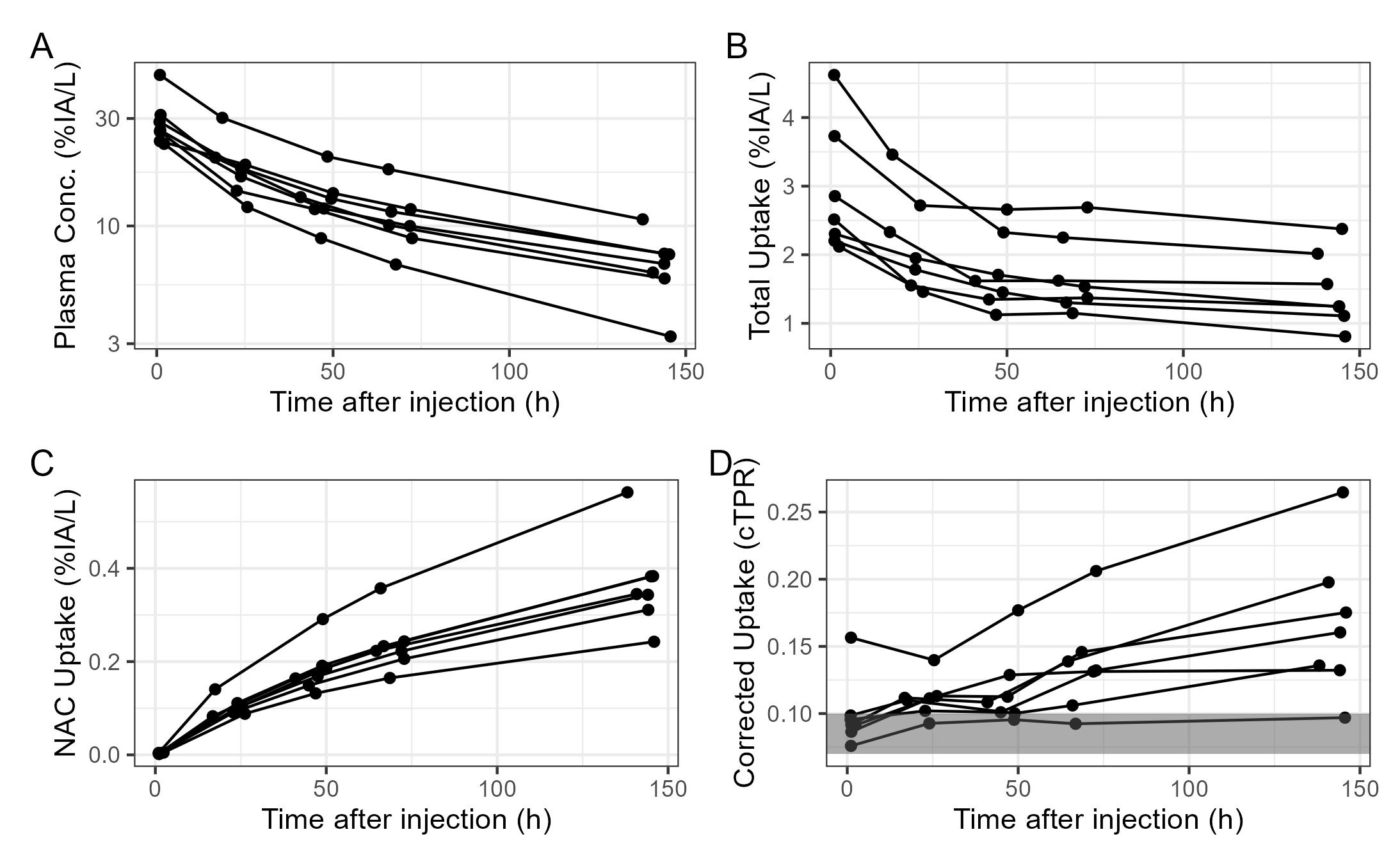


**Supplemental Figure 4**. Measured ^89^Zr-cetuximab concentration in plasma (panel A, in %IA/L), total measured ^89^Zr-cetuximab lung uptake (panel B, in TPR), calculated uptake due to nonspecific antibody catabolism of ^89^Zr-cetuximab in the lung (panel C, in TPR) and nonspecific antibody catabolism corrected ^89^Zr-cetuximab lung uptake (panel D, in cTPR). The gray area in panel D indicates the range of bTPR_lung_ values from Table 1. Each line represents data from a single patient.
